# 7α, 25-dihydroxycholesterol-mediated activation of EBI2 in immune regulation and diseases

**DOI:** 10.3389/fphar.2015.00060

**Published:** 2015-03-24

**Authors:** Siquan Sun, Changlu Liu

**Affiliations:** ^1^Immunology Therapeutic Area, Janssen Pharmaceutical Research & Development, LLC, San DiegoCA, USA; ^2^Neuroscience Therapeutic Area, Janssen Pharmaceutical Research & Development, LLC, San DiegoCA, USA

**Keywords:** EBI2, 7α, 25-OHC

## Abstract

EBI2, aka GPR183, is a G-couple receptor originally identified in 1993 as one of main genes induced in Burkitt’s lymphoma cell line BL41 by Epstein–Barr virus (EBV) infection. After it was reported in 2009 that the receptor played a key role in regulating B cell migration and responses, we initiated an effort in looking for its endogenous ligand. In 2011 we and another group reported the identification of 7α, 25-dihydroxyxcholesterol (7α, 25-OHC), an oxysterol, as the likely physiological ligand of EBI2. A few subsequently published studies further elucidated how 7α, 25-OHC bound to EBI2, and how a gradient of 7α, 25-OHC could be generated *in vivo* and regulated migration, activation, and functions of B cells, T cells, dendritic cells (DCs), monocytes/macrophages, and astrocytes. The identification of 7α, 25-OHC as a G protein-coupled receptor ligand revealed a previously unknown signaling system of oxysterols, a class of molecules which exert profound biological functions. Dysregulation of the synthesis or functions of these molecules is believed to contribute to inflammation and autoimmune diseases, cardiovascular diseases, neurodegenerative diseases, cancer as well as metabolic diseases such as diabetes, obesity, and dyslipidemia. Therefore EBI2 may represent a promising target for therapeutic interventions for human diseases.

## Introduction

G protein-coupled receptors (GPCRs), through ligation and activation by cognate ligands, regulate key cellular functions including generation, homeostasis, activation, differentiation, proliferation, migration. Functional dysregulation of these receptors often lead to pathogenesis in many human diseases. Therefore these receptors represent a class of highly druggable targets, which counted for a significant number of top marketed drugs in recent years (Andrews et al., 2014). Among ~800 predicted GPCR genes, over 600 belong to a largest group of Rhodopsin-like family (Class A) and about half of which are believed to be liganded receptors. For this group liganded receptors, there are still ~100 GPCR whose cognate ligands have not been identified, which are of high interest for discovering novel biological mechanisms and potential drug targets. At Janssen, our past efforts have de-orphanized a number of these orphans including H3, H4 (Lovenberg et al., 1999; Liu et al., 2001, 2003, 2005, 2009; Hannedouche et al., 2011).

EBI2, aka, GPR183, was originally identified in Birkenbach et al. (1993) as one of main genes induced in Burkitt’s lymphoma cell line BL41 by Epstein–Barr virus (EBV) infection. Gatto et al. (2009) and Pereira et al. (2009), two groups independently reported that the receptor played a key role in regulating B cell migration and responses, which prompted our interest and effort in looking for its endogenous ligand. Two years later, our group and the other (Hannedouche et al., 2011; Liu et al., 2011) independently discovered and reported the identification of 7α, 25-dihydroxycholesterol (7α, 25-OHC) as the endogenous ligand for EBI2. This was further supported by subsequent studies (Benned-Jensen et al., 2012; Zhang et al., 2012) detailing how 7α, 25-OHC binds and activates EBI2, and how the oxysterol is produced and how a gradient of 7α, 25-OHC is generated *in vivo* (Yi et al., 2012). Besides its role in regulating immune cell migration (**Table 1**), there were also published evidences linking EBI2 with human diseases, including type 1 diabetes (T1D; Heinig et al., 2010), multiple sclerosis (MS; Chalmin et al., 2015), and cancer cell proliferation (Benned-Jensen et al., 2011). Therefore EBI2 may represent a promising target for a number of indications such as inflammatory or autoimmune diseases, metabolism diseases, and cancer.

**Table 1:** Function of EBI2 in immune cells.

Cell types	Function of EBI2	Reference
B cells	Migration	Gatto et al. (2009, 2011), Pereira et al. (2009), Hannedouche et al. (2011), Liu et al. (2011)
	Enhanced proliferation	Benned-Jensen et al. (2011)
T cells	Migration	Hannedouche et al. (2011), Liu et al. (2011), Chalmin et al. (2015)
DC, monocyte	Migration	Hannedouche et al. (2011), Liu et al. (2011), Gatto et al. (2013), Yi and Cyster (2013), Gessier et al. (2014), Preuss et al. (2014)
	Negative regulation of type I interferon	Heinig et al. (2010), Chiang et al. (2013)
Astrocytes	Migration	Rutkowska et al. (2015)

In this review, we describe our de-orphanization effort leading to the identification of 7α, 25-OHC, summarize published data related to EBI2 receptor function and in human diseases, and small molecule EBI2 modulators reported to date.

## Role of EBI2 in B Cells Migration

Secondary lymphoid tissues such as spleen and lymph nodes consist of distinct functional compartments such as B cell follicles that surround a central T cells zone. In spleen, follicles are also surrounded by marginal zone where marginal zone innate B cells localize. Functional compartmentation of these tissues is controlled by spatial and temporal expression of chemokines by stromal or resident cells, and chemokine receptors by migratory lymphocytes including T cells, B cells, and DC. For example, naïve B cells express high level of CXCR5, home to follicles via gradient of CXCL13 produced by follicular stromal cells. Upon antigen encounter, activated B cells upregulate the expression of CCR7, and migrate toward T-B boundary in responding to CCL19 and CCL21 which are produced by cells within T cell zone. As B cells response progresses, differentiating B cells within germinal centers regulate expression of another receptor CXCR4, the receptor for CXCL12, which segregate B cells into light zone and dark zone, and also direct migration of plasma cells to splenic red pulp and bone marrow.

Gatto et al. (2009) and Pereira et al. (2009) two groups reported that EBI2 played a critical role in regulating B cell migration during immune activation. It appeared that naïve B cells express high level of EBI2, which was further upregulated upon B cell activation, and interestingly expression of EBI2 was significantly down-regulated in germinal center B cells. Employing B cells which were deficient or overexpressing EBI2, the authors demonstrated that increased expression of EBI2 directed activated B cell migration to outer follicular areas; and down-regulation of EBI2 was a prerequisite for migration into central follicular areas to form germinal centers. Although it appeared that EBI2 did not directly affect activation and proliferation of B cells, B cells deficient in EBI2 nonetheless led to defective antibody responses *in vivo*. These seminal findings clearly suggested EBI2 played a role in B cells migration. Therefore, together with CXCR5, CXCR4, and CCR7, EBI2 appeared to be another key chemotactic receptor to direct B cell migration and positioning within secondary lymphoid tissues. This notion was further demonstrated later by a study using compound knockout mice in which one, two or all three of receptors including CXCR5, CCR7, and EBI2 were knocked out (Gatto et al., 2011).

## De-orphanization of EBI2

In 2011, we and another group (Hannedouche et al., 2011; Liu et al., 2011), independently identified 7α, 25-OHC as the endogenous ligand for EBI2 and demonstrated that the oxysterol functioned as a chemotactic molecule for B cell migration.

To identify EBI2 ligand, the two groups took similar approaches starting from detecting EBI2-specific activity in partially purified rat, porcine, or sheep tissue extracts, followed by chromatography–mass spectrometry (GCMS) and NMR methods to identify the active molecule as an oxysterol. While Hannedouche et al. (2011) directly showed the final purified activity as 7α, 25-OHC, we took a slightly different approach. Once we identified certain EBI2 active oxysterols such as 7α-OHC, 7β-OHC, and 25-OHC through GCMS and NMR, we acquired and tested a number of commercially available oxysterols. The results led us to speculate that 7α, 25-OHC was likely the most potent EBI2 agonist, which was confirmed using a synthesized compound. We then further showed that starting from extract of a single mouse spleen, the only significant activity which could be purified and detected co-migrated in GCMS with the synthetic 7α, 25-OHC, and the identity of the active purified fraction was further confirmed by NMR.

**FIGURE 1:**
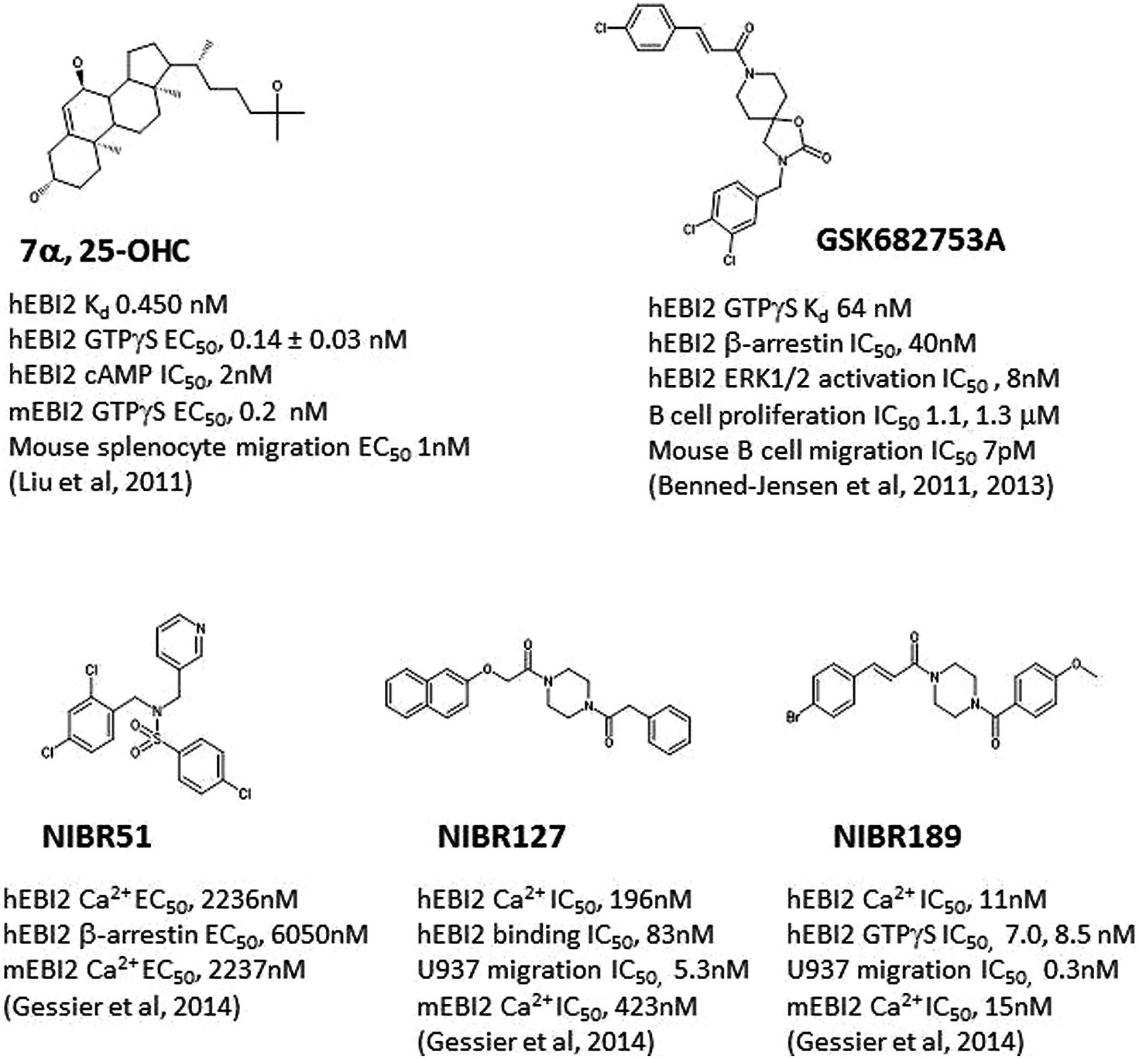
**Structures of selected E1312 agonists and antagonists, and their activities**. Key compound activities and associated publications were listed.

Both groups demonstrated that 7α, 25-OHC showed saturated binding to EBI2 in radioligand binding assays. 7α, 25-OHC was highly potent in binding and activating human EBI2 in cells (**Figure 1**; K_d_ 450 pM; GTPγS EC_50_ 140 pM), and coupled to G_i/o_ proteins (e.g., inhibition of cAMP production IC_50_, 2 nM). Consistent with previously findings indicting a role of EBI2 in driving B cell migration, 7α, 25-OHC induced migration of mouse and human B cells *in vitro*, and interestingly T cells and DC as well, in an EBI2-dependent manner. In cells, ligation of EBI2 by 7α, 25-OHC induced receptor internalization and desensitization. Thus, pretreatment of mouse B cells with 7α, 25-OHC for 1 h *in vitro* rendered these cells refractory to migration induced by 7α, 25-OHC, and interestingly a few other chemokines such as CXCL12, CXCL13, and CCL19/21 as well.

Further experiments showed that the expression of EBI2 and also the two enzymes required for synthesis of 7α, 25-OHC biosynthesis, i.e., cholesterol 25-hydrxylase (Ch25H) and oxysterol 7α-hydroxylase (Cyp7B1) was highly modulated by inflammatory stimuli such as LPS in various immune cells such as B cells, T cells, and dendritic cells (DCs) *in vitro* as well as *in vivo* in mice. In a B cell adoptive transfer model, we showed that 7α, 25-OHC-pretreatment of B cells (therefore desensitized) reduced homing of these cells into follicles after adoptive transfer, suggesting EBI2 also plays a role in B cell homing to secondary lymphoid tissues.

To demonstrate the role of 7α, 25-OHC *in vivo*, we employed a model in which host mice were dosed with Clotrimazole, an anti-fungi compound with known Cyp7B1 inhibition activity which was shown to significantly reduce level of 7α, 25-OHC *in vitro* and *in vivo*. In comparison, Hannedouche et al. (2011) employed Ch25H KO mice as hosts which as expected were deficient in production of 7α, 25-OHC. In both models adoptively transferred wild-type B cells showed identical migration pattern as those for Ebi2-deficient B cells adoptively transferred into vehicle-treated or wild-type hosts. Thus, for example, our studies showed that adoptively transferred wild-type B cells migrated into and localized throughout splenic follicles in normal hosts; however, in Clotrimazole-treated hosts, a much greater percentage of transferred B cells was localized at the T-B boundary, presumably due to CCR7-dependent migration of these B cells and lack of EBI2-driven migration at the same time. In addition, Hannedouche et al. (2011) further showed that, as a functional consequence, plasma cell response in Ch25H KO mice was similarly defective as that in Ebi2-deficient mice.

A subsequent study by Yi et al. (2012) suggested that lymphoid stromal cells were the main Ch25H- and Cyp7B1-expressing cells and were responsible for production of 7α, 25-OHC *in vivo*. In addition, the group also demonstrated that the same cells were likely also responsible for degradation of 7α, 25-OHC by expressing HSD3B7 which converts 7α, 25-OHC into a 3-oxo derivative. Importantly, deficiency in any three of these enzymes affected B cell positioning *in vivo*, and resulted in defective T cell-dependent plasma cell response.

Taken together, the data from these studies clearly demonstrated that 7α, 25-OHC was the endogenous EBI2 ligand driving B cells migration, and modulating B cell antibody responses *in vivo*.

## Binding of 7α, 25-OHC to EBI2

Since EBI2 is the first GPCR known to bind and be activated by an oxysterol, we carried out and later published (Zhang et al., 2012) a study to understand the molecular and structural basis for its ligand-dependent activation.

Using a site-directed mutagenesis approach, we generated mutated receptors that carried single amino acid substitutions. The pharmacological profiles of these mutant receptors were characterized by measuring receptor expression, radioligand binding, and receptor activation. Within the highly variable extracellular regions, we compared sequences from human, rat and mouse, and selected a number of conserved residues for mutagenesis study. This led to identification of Glu183 in ECL2, Ala-substitution of which resulted in significant reduction of ligand binding and ~50-fold potency reduction.

Transmembrane (TM) regions of GPCR are structurally conserved and the helical cores are thought to make up the ligand binding pocket. We aligned EBI2 TM sequences with those of GPCR with a defined ligand binding pocket, and selected key residues for mutagenesis analysis. Among key residues identified by these analyses, Arg87 (TM2) to Ala, or Trp and Asn114 to Ala resulted in diminished receptor binding and ~500 fold or more reduction in ligand potency. A number of other residues that showed reduced ligand binding and receptor function after Ala substitution included Tyr112 and Tyr116 of TM3; Leu197 of TM5; Tyr260, His216, and Ile264 of TM6; Val294 and M297 of TM7. Moreover, Ala substitution of Asp77 showed no effect on ligand binding, however, was found to severely reduce receptor activation suggesting the residue was important for receptor activation or signal transduction.

By examining various analogs of 7α, 25-OHC in EBI2-dependent assays, we showed that all three hydroxyl groups at 3′, 7′α, and 25′ were required for receptor binding and activation. Using a hybrid β2AR-CXCR4 structure as a template, we created a homology model for EBI2. Optimization of docking of 7α, 25-OHC into the putative ligand binding pocket of the EBI2 model structure suggested that residues Arg87, Asn114, and Glu183 interact with 7′α-, 25′-, and 3′-hydroxyl group respectively.

In addition, our analysis also suggested that Cys104 (ECL1) and Cys181 (ECL2) form the classic disulfide bond observed in many other GPCR, while Cys21 (N-terminal) and Cys280 (ECL3) may form a second disulfide bridge. This pattern of extracellular disulfide bridges appeared to be the same as in CXCR4. This proposed model of two disulfide bridges has important functional implications. Because of the Cys104-Cys181 link, ECL2 is brought close to the TM domain and may form a cap over the putative ligand binding site. As a result, Glu183 with ECL2 is positioned within the ligand binding domain, representing the best candidate forming interaction with 3′-hydroxyl group of 7α, 25-OHC. This notion was further supported by the inactivity of 7α, 27-dihydroxy-4-cholesten-3-one where a ketone interacting with the carboxylate would be energetically unfavorable.

Another group (Benned-Jensen et al., 2012) published a similar mutagenesis study around the same time. In that study, the authors focused on residues facing the main putative ligand binding pocket for mutagenesis analysis, which led to identification of four key residues required for 7α, 25-OHC binding: Arg87, Tyr112 and Tyr116, and Tyr260. These findings are consistent with what we reported. Docking modeling by these authors suggested a different model from ours, i.e., Y116 interacts with 3′-hydroxyl group, Y260 with 7′α-hydroxyl group, and Y260 directly or indirectly with 25′-hydroxyl group.

Together, these two studies started to shed some insights on how an oxysterol ligand could bind and activate a GPCR, which may potentially benefit discovery of novel EBI2 modulators.

## Role of EBI2 in Immune Responses and Diseases

It is now clear that EBI2 plays important roles in modulating activation and controls migration of various immune cells (**Table 1**). The function of EBI2 in B cells *in vivo* appears to be largely dependent on directing B cell migration and positioning in secondary lymphoid tissues. As described above, EBI2 deficiency or deficiency in 7α, 25-OHC biosynthesis resulted in reduce plasma cell responses. *In vitro*, EBI2-deficient mouse B cells did not show overt deficiency in activation including proliferation or upregulation of cell surface marker such as CD86 (Gatto et al., 2009). Neither did we observe much effect with 7α, 25-OHC when added to B cell cultures (unpublished data). However, it was reported that B cells from transgenic mice with overexpression of human EBI2 showed enhanced antibody-induced proliferation (Benned-Jensen et al., 2011). Therefore, it remains a possibility that EBI2 ligation may directly affect certain aspect of B cell activation. To this end, it was speculated that dysregulation of EBI2 expression might contribute in B cell malignancies (Benned-Jensen et al., 2011, 2013). For example, EBI2 expression was increased in post-transplantation lymphoproliferative diseases (PTLDs; Craig et al., 2007). Therefore, it was proposed that an EBI2 antagonist might offer therapeutic benefit in blocking proliferation of EBV-transformed B cell lymphomas (Benned-Jensen et al., 2011, 2013).

In addition to B cells, EBI2 was also found to be expressed in T cells, and DC/monocytes (Hannedouche et al., 2011; Liu et al., 2011). These findings suggested that EBI2 may play key roles in regulating other immune cells. *In vitro*, 7α, 25-OHC clearly induced migration of mouse and human T cells and CD11c^+^ DC/monocytes. One recent report (Chalmin et al., 2015) showed that Ch25h deficiency led to significantly attenuated disease in mouse experimental autoimmune encephalomyelitis (EAE). a model of MS, The authors showed that there was a critical involvement for oxysterols in recruiting leukocytes into the inflamed central nervous system (CNS) and proposed that 7α, 25-OHC preferentially promoted EBI2-dependent migration of activated CD44^+^CD4^+^ T cells. In a related study (Wanke et al., 2014), using a novel reporter-knockin/knockout mouse model, the authors showed that pathogenic Th1 and Th17 cells expressed EBI2 within inflamed CNS, while T_reg_ cells showed a bipartite expression. Using a transfer model of EAE, the authors showed that EBI2-deficient Th17 cell displayed a significantly delayed pathogenesis compared to wild-type Th17 cells. Taken together, these studies suggested EBI2-dependent migration of T cells might play a role in pathogenesis in autoimmune disease such as MS.

EBI2 also appeared to play an important role in controlling DC migration and homeostasis in mice. Two groups (Gatto et al., 2013; Yi and Cyster, 2013) independently reported that mouse splenic CD4^+^ DC highly expressed EBI2 and migrated in response to 7α, 25-OHC. In mice which were deficient in EBI2 or 7α, 25-OHC biosynthesis, there was a reduced frequency of splenic CD4^+^ DC number. The authors further showed that the deficiency was likely due to defective CD4^+^ DC migration as there was defective positioning of remaining CD4^+^ DC to marginal zone bridging channels and EBI2 did not affect development of DC or DC survival. As one of the main functions of CD4^+^ DC in the marginal zone bridging channels was to promote DC encounter of blood-borne particulate antigens, EBI2 deficiency in these specific DC led to defects in both activation of helper T cells and induction of antibody response to this type of antigens.

Expression of EBI2, Ch25H, and Cyp7B1 was found to be highly unregulated in a number of inflammatory diseases, including chronic rhinosinusitis (CRS; Hulse et al., 2013). Nasal polyps from CRS patients showed increased expression of EBI2 which was positively correlated with the expression of plasma cell markers (such as CD138 and B lymphocyte-induced maturation protein) in sinus tissue. Upregulation of Cyp7B1 was observed in inflamed joints in collagen-induced arthritis model (Dulos et al., 2004), a mouse model of rheumatoid arthritis (RA). The same findings applied to fibroblast-like synoviocytes obtained from RA synovial tissues (Dulos et al., 2005), which suggested that EBI2 might also play a role in RA. Interestingly, supporting this notion, the CYP7B1 inhibitor Clotrimazole was tested in RA and showed some efficacy (Wojtulewski et al., 1980).

Human primary macrophages were also reported (Preuss et al., 2014) to express EBI2, Ch25H, and Cyp7B1. In cultured monocyte-derived macrophages, LPS triggered a strong up-regulation of Ch25H and Cyp7B1 and a transient increase in EBI2 expression. Ligation of EBI2 in cultured macrophages led to calcium mobilization and cell migration. Culture supernatants of LPS-stimulated macrophages were able to activate EBI2 signaling indicating that these cells produced 7α, 25-OHC upon LPS-induction of Ch25H and Cyp7B1.

A genome-wide association study using both rat and human data published in Heinig et al. (2010) implicated EBI2 as a regulator of an interferon regulatory factor 7 (IRF)-driven inflammatory network (IDIN), which was associated with susceptibility of T1D. The human locus controlling IDIN was associated with the risk of T1D at a single nucleotide polymorphism rs9585056, which was also associated with increased EBI2 expression. siRNA knockdown of EBI2 in rat macrophages increased expression of Irf7 and IDIN genes. Another study (Chiang et al., 2013) demonstrated that EBI2 negatively regulated type I interferon (IFN) responses in plasmacytoid DC and CD11b^+^ myeloid cells. EBI2 deficiency led to increased production of type I interferon induced by TLR ligands *in vitro* as well as *in vivo*. Therefore, it will be interesting to explore whether dysregulation of EBI2 plays a role in pathogenesis of certain autoimmune disease such as lupus, Sjogren’s syndrome in which type I interferon is implicated (Swiecki and Colonna, 2011). Oxysterols and oxysterol sensing receptors have been shown to modulate metabolism (Gill et al., 2008; Raychaudhuri and Prinz, 2010) and may play roles in metabolic disease such as diabetes, obesity, and dyslipidemia. The link between EBI2 and T1D suggested that EBI2 may also have a similar role.

One group (Rutkowska et al., 2015) recently showed that human and mouse astrocytes express EBI2 and the enzymes necessary for synthesis and degradation of 7α, 25-OHC. In astrocytes, EBI2 activation stimulates ERK phosphorylation, Ca^2+^ signaling and induces cellular migration. In addition, activation EBI2 prevents lysophosphatidylcholine (LPC)-induced demyelination in cerebellar slices. These results suggested that EBI2 is involved in glial cell function and modulation of this receptor may be beneficial in neuroinflammatory or neurodegenerative disorders.

## EBI2 Modulators

As described above, it has been demonstrated that EBI2 serves as a key chemotactic receptor for B cells, T cells and DC, modulating both T cell and B cell response to blood-borne antigens. The expression of EBI2 and enzymes for 7α, 25-OHC is upregulated in inflammatory diseases, and dysregulation of EBI2 may contribute to B cell malignancies, and certain autoimmune diseases such as T1D, RA, lupus, and MS. Therefore it is of interest to discover and develop EBI2 modulators and test for their therapeutic potentials in preclinical models and in patients.

Simultaneous to discovery of 7α, 25-OHC as the endogenous EBI2 ligand, one group (Benned-Jensen et al., 2011) reported the identification and characterization of a small molecule inverse agonist, GSK682753A (**Figure 1**), which blocked the apparent constitutive activity of EBI2 receptor in a recombinant system. It was later (Benned-Jensen et al., 2013) shown that the compound was actually a potent EBI2 antagonist, blocking 7α, 25-OHC induced EBI2 activation *in vitro*. A number of compounds were also identified using a uHTS screen at the Sanford-Burnham Center for Chemical Genomics which inhibited 7α, 25-OHC-mediated β-arrestin recruitment (PubChem BioAssay ID: 651636).

Another group (Gessier et al., 2014) recently reported the identification of a small molecule agonist NIBR51 (**Figure 1**; hEBI2 EC_50_ ~2 μM) from screening about 100 K compounds in an EBI2-dependent recombinant assay. Using this agonist, the group rescreened the same library and identified a small molecule antagonists NIBR127 (**Figure 1**) which bind to EBI2 with high affinity (human EBI2 EC_50_ 83 nM) and good potency in receptor activation (hEBI2 Ca^2+^ IC_50_ 196 nM). A subsequent medicinal chemistry effort using NIBR127 as a starting point led to NIBR189 (**Figure 1**) with significant improved potency in EBI2 receptor activation (hEBI2 Ca^2+^ IC_50_ 11 nM). Further pharmacological characterization suggested that NIBR189 was a potent and selective EBI2 antagonist with pharmacokinetic properties which should allow for *in vitro* and *in vivo* experiments. To this end, it was noted by the authors that much of initial testing was directed toward paradigms of autoimmune disorders. Because of the demonstrated activity of EBI2 in monocytes as described above, it was proposed by the authors to test the EBI2 antagonist in paradigms relevant for cardiovascular diseases (Gessier et al., 2014).

## Concluding Remarks

The identification of 7α, 25-OHC as the endogenous EBI2 ligand uncovered a previously unknown role for oxysterols, a class of molecules which exert profound biological effects. Work published by a number of groups including ours in last few years showed that EBI2 and key enzymes in producing 7α, 25-OHC were highly regulated during inflammation, and might play key roles in pathogenesis of certain human diseases such as to inflammation and autoimmune diseases, neurodegenerative disease, cardiovascular diseases, cancers, as well as metabolic diseases such as diabetes, obesity, and dyslipidemia. Therefore EBI2 may represent a promising target for therapeutic interventions.

## Conflict of Interest Statement

The authors declare that the research was conducted in the absence of any commercial or financial relationships that could be construed as a potential conflict of interest.

## References

[B1] Andrews S. P. Brown G. A. Christopher J. A. (2014), Structure-based and fragment-based GPCR drug discovery. *Chem. Med. Chem.* 9 256–275 10.1002/cmdc.201300382 24353016

[B2] Benned-Jensen T. Madsen C. M. Arfelt K. N. Smethurts C. Blanchard A. Jepras R. (2013). Small molecule antagonism of oxysterol-induced Epstein–Barr virus induced gene 2 (EBI2) activation. *FEBS Open Bio*. 3 156–160 10.1016/j.fob.2013.02.003 PMC3668520 23772388

[B3] Benned-Jensen T. Norn C. Laurent S. Madsen C. M. Larsen H. M. Arfelt K. N. (2012). Molecular characterization of oxysterol binding to the Epstein–Barr virus-induced gene 2 (GPR183). *J. Biol. Chem.* 287 35470–35483 10.1074/jbc.M112.387894 22875855 PMC3471686

[B4] Benned-Jensen T. Smethurst C. Holst P. J. Page K. R. Sauls H. Sivertsen B. (2011). Ligand modulation of the Epstein–Barr virus-induced seven-transmembrane receptor EBI2: identification of a potent and efficacious inverse agonist *J. Biol. Chem.* 2011 286 29292–29302 10.1074/jbc.M110.196345 21673108 PMC3190735

[B5] Birkenbach M. Josefsen K. Yalamanchili R. Lenoir G. Kieff E. (1993). Epstein–Barr virus-induced genes: first lymphocyte-specific G protein-coupled peptide receptors. *J. Virol.* 67 2209–2220. 8383238 10.1128/jvi.67.4.2209-2220.1993 PMC240341

[B6] Chalmin F. Rochemont V. Lippens C. Clottu A. Sailer A. W. Merkler D. (2015). Oxysterols regulate encephalitogenic CD4(+) T cell trafficking during central nervous system autoimmunity. *J. Autoimmun.* 56 45–55 10.1016/j.jaut.2014.10.001 25456971

[B7] Chiang E. Y. Johnston R. J. Grogan J. L. (2013). EBI2 Is a negative regulator of type I interferons in plasmacytoid and myeloid dendritic cells. *PLoS ONE* 8:e83457 10.1371/journal.pone.0083457 PMC3873289 24386204

[B8] Craig F. E. Johnson L. R. Harvey S. A. Nalesnik M. A. Luo J. H. Bhattacharya S. D. (2007). Gene expression profiling of Epstein–Barr virus-positive and negative monomorphoic B-cell posttransplant lymphoproliferative disorders. *Diagn. Mol. Pathol.* 16 158–168 10.1097/PDM.0b013e31804f54a9 17721324

[B9] Dulos J. van der Vleuten M. A. Kavelaars A. Heijnen C. J. Boots A. M. (2005). CYP7B expression and activity in fibroblast-like synoviocytes from patients with rheumatoid arthritis: regulation by proinflammatory cytokines. *Arthritis Rheum.* 52 770–778 10.1002/art.20950 15751070

[B10] Dulos J. Verbraak E. Bagchus W. M. Boots A. M. Kaptein A. (2004). Severity of murine collagen-induced arthritis correlates with increased CYP7B activity: enhancement of dehydroepiandrosterone metabolism by interleukin-1beta. *Arthritis Rheum.* 50 3346–3353 10.1002/art.20509 15476247

[B11] Gatto D. Paus D. Basten A. Mackay C. R. Brink R. (2009). Guidance of B cells by the orphan G protein-coupled receptor EBI2 shapes humoral immune responses. *Immunity* 31 1–11 10.1016/j.immuni.2009.06.016 19615922

[B12] Gatto D. Wood K. Brink R. (2011). EBI2 operates independently of but in cooperation with CXCR5 and CCR7 to direct B cell migration and organization in follicles and the germinal center. *J. Immunol.* 187 4621–4628 10.4049/jimmunol.1101542 21948984

[B13] Gatto D. Wood K. Caminschi I. Murphy-Durland D. Schofield P. Christ D. (2013). The chemotactic receptor EBI2 regulates the homeostasis, localization and immunological function of splenic dendritic cells. *Nat. Immunol.* 14 446–453 10.1038/ni.2555 23502855

[B14] Gessier F. Preuss I. Yin H. Rosenkilde M. M. Laurent S. Endres R. (2014), Identification and characterization of small molecule modulators of the Epstein–Barr virus-induced gene 2 (EBI2) receptor. *J. Med. Chem*. 57 3358–3368 10.1021/jm4019355 24678947

[B15] Gill S. Chow R. Brown A. J. (2008). Sterol regulators of cholesterol homeostasis and beyond: the oxysterol hypothesis revisited and revised. *Prog.* *Lipid Res.* 2008 47 391–404 10.1016/j.plipres.2008.04.002 18502209

[B16] Hannedouche S. Zhang J. Yi T. Shen W. Nguyen D. Pereira J. P. (2011). Oxysterols direct immune cell migration via EBI2. *Nature* 475 524–527 10.1038/nature10280 21796212 PMC4297623

[B17] Heinig M. Petretto E. Wallace C. Bottolo L. Rotival M. Lu H. (2010). A trans-acting locus regulates an anti-viral expression network and type 1 diabetes risk. *Nature* 467 460–464 10.1038/nature09386 20827270 PMC3657719

[B18] Hulse K. E. Norton J. E. Suh L. Zhong Q. Mahdavinia M. Simon P. (2013). Chronic rhinosinusitis with nasal polyps is characterized by B-cell inflammation and EBV-induced protein 2 expression. *J. Allergy Clin. Immunol.* 131 1075–1083 10.1016/j.jaci.2013.01.043 23473835 PMC3741659

[B19] Liu C. Eriste E. Sutton S. Chen J. Roland B. Kuei C. (2003). Identification of relaxin-3/INSL7 as an endogenous ligand for the orphan G-protein-coupled receptor GPCR135. *J. Biol. Chem.* 278 50754–50764 10.1074/jbc.M308995200 14522968

[B20] Liu C. Kuei C. Sutton S. Chen J. Bonaventure P. Wu J. (2005). INSL5 is a high affinity specific agonist for GPCR142 (GPR100). *J. Biol. Chem.* 280 292–300 10.1074/jbc.M409916200 15525639

[B21] Liu C. Ma X. J. Jiang X. Wilson S. J. Hofstra C. L. Blevitt J. (2001). Cloning and pharmacological characterization of a fourth histamine receptor (H4) expressed in bone marrow. *Mol. Pharmacol.* 59:420–426 10.1124/mol.59.3.420 11179434

[B22] Liu C. Wu J. Zhu J. Kuei C. Yu J. Shelton J. (2009). Lactate inhibits lipolysis in fat cells through activation of an orphan G-protein-coupled receptor, GPR81. *J. Biol. Chem.* 284 2811–2822 10.1074/jbc.M806409200 19047060

[B23] Liu C. Yang X. V. Wu J. Kuei C. Mani N. S. Zhang L. (2011). Oxysterols direct B-cell migration through EBI2. *Nature* 475 519–523 10.1038/nature10226 21796211

[B24] Lovenberg T. W. Roland B. L. Wilson S. J. Jiang X. Pyati J. Huvar A. (1999). Cloning and functional expression of the human histamine H3 receptor. *Mol. Pharmacol.* 55 1101–1107. 10347254

[B25] Pereira J. P. Kelly L. M. Xu Y. Cyster J. G. (2009), EBI2 mediates B cell segregation between the outer and centre follicle. *Nature* 460 1122–1126 10.1038/nature08226 19597478 PMC2809436

[B26] Preuss I. Ludwig M. G. Baumgarten B. Bassilana F. Gessier F. Seuwen K. (2014). Transcriptional regulation and functional characterization of the oxysterol/EBI2 system in primary human macrophages. *Biochem. Biophys. Res. Commun.* 446 663–668 10.1016/j.bbrc.2014.01.069 24480442

[B27] Raychaudhuri S. Prinz W. A. (2010). The diverse functions of oxysterol-binding proteins. *Annu. Rev. Cell Dev. Biol.* 26 157–177 10.1146/annurev.cellbio.042308.113334 19575662 PMC3478074

[B28] Rutkowska A. Preuss I. Gessier F. Sailer A. W. Dev K. K. (2015), EBI2 regulates intracellular signaling and migration in human astrocyte. *Glia* 63 341–351 10.1002/glia.22757 25297897

[B29] Swiecki M. Colonna M. (2011). Type I interferons: diversity of sources, production pathways and effects on immune responses. *Curr. Opin. Virol.* 1 463–475 10.1016/j.coviro.2011.10.026 22440910 PMC3572907

[B30] Wanke F. Croxford A. L. Heinen A. P. Firmenich S. Moos S. Israel N. (2014) Expression of the G-protein coupled receptor EBI2 in T cells is highly regulated and confers pathogenicity to myelin specific Th17 cells. *J. Neuroimmunol*. 275:211 10.1016/j.jneuroim.2014.08.566

[B31] Wojtulewski J. A. Gow P. J. Walter J. Grahame R. Gibson T. Panayi G. S. (1980). Clotrimazole in rheumatoid arthritis. *Ann. Rheum. Dis.* 39 469–472 10.1136/ard.39.5.469 7002065 PMC1000587

[B32] Yi T. Cyster J. G. (2013). EBI2-mediated bridging channel positioning supports splenic dendritic cell homeostasis and particulate antigen capture. *eLife* 2:e00757 10.7554/elife.00757 PMC3654440 23682316

[B33] Yi T. Wang X. Kelly L. M. An J. Sailer A. W. Gustafsson J. A. (2012). Oxysterol gradient generation by lymphoid stromal cells guides activated B cell movement during humoral responses. *Immunity* 37 535–548 10.1016/j.immuni.2012.06.015 22999953 PMC3465460

[B34] Zhang L. Shih A. Y. Yang X. V. Kuei C. Wu J. Deng X. (2012). Identification of structural motifs critical for Epstein–Barr virus-induced molecule 2 function and homology modeling of the ligand docking site. *Mol. Pharmacol.* 82 1094–1103 10.1124/mol.112.080275 22930711

